# Effects of HfB_2_ and HfN Additions on the Microstructures and Mechanical Properties of TiB_2_-Based Ceramic Tool Materials

**DOI:** 10.3390/ma10050461

**Published:** 2017-04-27

**Authors:** Jing An, Jinpeng Song, Guoxing Liang, Jiaojiao Gao, Juncai Xie, Lei Cao, Shiying Wang, Ming Lv

**Affiliations:** 1 School of Mechanical Engineering, Taiyuan University of Technology, Taiyuan 030024, China; anjing@tyut.edu.cn (J.A.); liangguoxing@tyut.edu.cn (G.L.); gaojiaojiao1207@163.com (J.G.); 15834068972@163.com (J.X.); 17603410780@163.com (L.C.); wangshiying@tyut.edu.cn (S.W.); lvming@tyut.edu.cn (M.L.); 2 Shanxi Key Laboratory of Precision Machining, The Shanxi Science and Technology Department, Taiyuan University of Technology, Taiyuan 030024, China

**Keywords:** TiB_2_-HfB_2_ ceramics, TiB_2_-HfN ceramics, hot-pressed sintering, microstructure, mechanical properties

## Abstract

The effects of HfB_2_ and HfN additions on the microstructures and mechanical properties of TiB_2_-based ceramic tool materials were investigated. The results showed that the HfB_2_ additive not only can inhibit the TiB_2_ grain growth but can also change the morphology of some TiB_2_ grains from bigger polygons to smaller polygons or longer ovals that are advantageous for forming a relatively fine microstructure, and that the HfN additive had a tendency toward agglomeration. The improvement of flexural strength and Vickers hardness of the TiB_2_-HfB_2_ ceramics was due to the relatively fine microstructure; the decrease of fracture toughness was ascribed to the formation of a weaker grain boundary strength due to the brittle rim phase and the poor wettability between HfB_2_ and Ni. The decrease of the flexural strength and Vickers hardness of the TiB_2_-HfN ceramics was due to the increase of defects such as TiB_2_ coarse grains and HfN agglomeration; the enhancement of fracture toughness was mainly attributed to the decrease of the pore number and the increase of the rim phase and TiB_2_ coarse grains. The toughening mechanisms of TiB_2_-HfB_2_ ceramics mainly included crack bridging and transgranular fracture, while the toughening mechanisms of TiB_2_-HfN ceramics mainly included crack deflection, crack bridging, transgranular fracture, and the core-rim structure.

## 1. Introduction

In recent years, with the widespread use of difficult-to-machine materials in engineering, cutting tools have faced the challenge of machining these materials under high speed, which requires that the tools have high hardness, excellent wear resistance, oxidation resistance, and so on. However, compared with the ceramic tool materials, the traditional tool materials (high-speed steel and cemented carbide) showed a lower red hardness in machining these difficult-to-machine materials, which did not meet the need of high speed machining. Recently, ceramic tools—Al_2_O_3_-based, Si_3_N_4_-based, and TiB_2_-based ceramic tools—exhibited excellent cutting performance in machining the difficult-to-machine materials such as martensitic stainless steel, Inconel 718, ultra-high-strength steel 300 M, heat-treated AISI4140, hardened Cr12MoV mold steel, and Invar36 alloy [1,2,3,4,5,6]. The TiB_2_-based ceramic tool exhibited higher hardness compared with the other ceramic tools, which was attributed to the higher hardness of TiB_2_ ceramic than that of the other ceramics. TiB_2_ also has a high melting point, excellent wear resistance, and oxidation resistance, which can also be applied in other fields such as manufacturing armor plates and dies [7,8,9,10]. However, it shows a tendency toward low flexural strength and low fracture toughness, which limits the more widespread application of TiB_2_. In order to reverse this tendency and improve the mechanical properties of TiB_2_ ceramic, reinforcements such as hard phases, metal phases, and whiskers have been employed to fabricate TiB_2_-based ceramic materials through spark plasma sintering, vacuum hot-pressed sintering, or reactive hot-pressed sintering. 

Usually, the hard phases included TaC, TiSi_2_, Al_2_O_3_, WC, TiC, B_4_C, NbC, MoSi_2_, SiC, and ZrB_2_ [9,10,11,12,13,14,15,16], which could inhibit the grain growth of the base material to obtain a fine microstructure. HfB_2_ and HfN have high hardness, high melting point, and high oxidation resistance, and as reinforcements they can enhance the mechanical properties of ceramics such as ZrB_2_-CrSi_2_-HfB_2_, ZrB_2_-SiC-HfB_2_, B_4_C-HfB_2_, and SiBCN-HfN [17,18,19,20], which make them potential candidate reinforcements for ceramic tool materials. In addition, because HfB_2_ and HfN have better thermal stability to resist deformation and decomposition at elevated temperature, they may improve the cutting performance and working life of TiB_2_-based ceramic tools. The metal phases often contain Fe, Co, Ni, and Mo [7,12,21,22], which could decrease the sintering temperature and improve the boundary strength among grains and relative density, while the ceramic whiskers such as aluminum borate whiskers and SiC whiskers could change the direction of crack growth to consume more crack propagation energy [23,24,25], which could improve the flexural strength and fracture toughness. Usually, adopting a combination of reinforcements for fabricating TiB_2_-based ceramics can obtain better mechanical properties. In addition, compared with spark plasma sintering that is employed in fabricating the ceramic composites [9,16], vacuum hot-pressed sintering is considered to be easily adaptable and economically viable.

In this paper, TiB_2_-HfB_2_ and TiB_2_-HfN ceramic tool materials will be fabricated with powders of TiB_2_, HfB_2_, HfN, Mo, and Ni by vacuum hot-pressed sintering. The characteristics of these composites are analyzed according to their microstructures and mechanical properties.

## 2. Experimental Procedures

Commercially available TiB_2_ powder (99.9%, 1 μm, Shanghai Xiangtian Nanomaterials Co., Ltd., Shanghai, China), HfB_2_ powder (99.9%, 0.8 μm, Shanghai Chaowei Nanomaterials Co., Ltd., Shanghai, China) and HfN powder (99.9%, 0.8 μm, Shanghai Chaowei Nanomaterials Co., Ltd.) were used as the raw materials. Ni powder (99.8%, 1 μm, Shanghai Yunfu Nanotechnology Co., Ltd., Shanghai, China) and Mo powder (99.8%, 1 μm, Shanghai Yunfu Nanotechnology Co., Ltd.) were added as sintering aids. The compositions of the composite tool materials are shown in Table 1.

The powders were mixed and milled for 48 h in a polyethylene jar with WC (tungsten carbide) balls and alcohol as the medium. Then the mixed slurry was dried in vacuum and sieved by a 200-mesh sieve. The compacted powders were hot pressed for 30 min at 1650 °C under 30 MPa in a vacuum ((1.2–2.4) × 10^−3^ Pa). The hot pressed samples were cut into testing specimens by the electrical discharge wire cutting method and the surfaces of the testing bars were polished using diamond slurries. The dimensions of the specimens were 3 mm × 4 mm × 40 mm.

Flexural strength was measured at a span of 30 mm and a crosshead speed of 0.5 mm/min by the three-point bending test method on an electron universal tester (CREE-8003G, Dongguan City Kerry Instrument Technology Co., Ltd., Dongguan, China), according to Chinese National Standards GB/T 6569-2006/ISO 14704:2000 [26]. The fracture toughness (*K_IC_*) was measured via the direct indentation method and was calculated through the following equation [12,27]:

$$K_{IC}=0.203H_{V}a^{1/2}{\left(\frac{c}{a}\right)}^{-3/2}$$

where *H_V_* is the Vickers hardness, 2*a* is the length of the impression diagonal, and 2*c* is the overall indentation crack length including 2*a*. The indenter (HVS-30, Shanghai Precision Instruments Co., Ltd., Shanghai, China) was of Vickers DPH (diamond pyramid hardness) type and the applied static load was 196 N for 15 s. Vickers hardness was measured on the polished surfaces using a diamond pyramid indenter under a load of 196 N by an HV-120 based on Chinese National Standards GB/T 16534-2009 [28]. The relative density of each specimen was measured by the Archimedes method with distilled water as the medium. The theoretical density was calculated according to the rule of mixtures based on the following densities: 4.52, 10.50, 13.80, 8.90, and 10.20 g/cm^3^ for TiB_2_, HfB_2_, HfN, Ni, and Mo, respectively. At least 15 specimens were tested for each experimental condition. X-ray diffraction (XRD, EMPYREAN, PANalytical B.V., Almelo, Netherlands) and energy dispersive spectrometry (EDS, ACT-350, Oxford Instruments, Oxford, UK) were used to analyze the compositions of the composite. Scanning electron microscopy and back scattered electron microscopy (SEM, BSE, Supra-55, Carl Zeiss AG, Oberkochen, Germany) were used to observe the polished surface and fractured surface morphologies.

## 3. Results and Discussions

### 3.1. Microstructure

**Figure 1:**
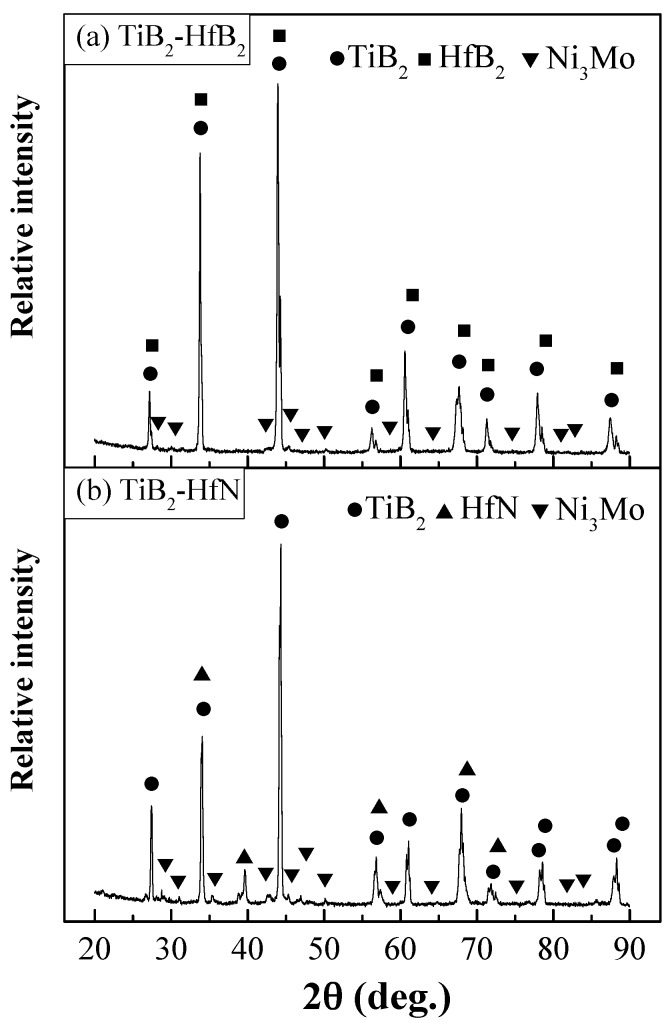
XRD patterns of TiB_2_-HfB_2_ and TiB_2_-HfN ceramic tool materials.

Figure 1 shows the XRD patterns of the TiB_2_-HfB_2_ and TiB_2_-HfN ceramic tool materials. The major crystal phases are TiB_2_ and HfB_2_ in the TiB_2_-HfB_2_ ceramics, and TiB_2_ and HfN in the TiB_2_-HfN ceramics. The minor phase is the Ni_3_Mo intermetallic compound in the TiB_2_-HfB_2_ and TiB_2_-HfN ceramic tool materials. This is because Ni and Mo can form the Ni_3_Mo intermetallic compound at 1300 °C [29]. The Ni_3_Mo intermetallic compound has a high melting point of about 1320 °C, so it may be a promising high-temperature structural material [30]. Compared with the standard peaks, the peaks of HfB_2_ are offset about two degrees to the right and are near the peaks of TiB_2_. This indicates there is likely an exchange of Ti and Hf atoms in the sintering, which leads to a complex solid solution of TiB_2_ and HfB_2_ formed in the ceramic tool materials. The peaks of HfN are in accordance with the standard peaks.

**Figure 2:**
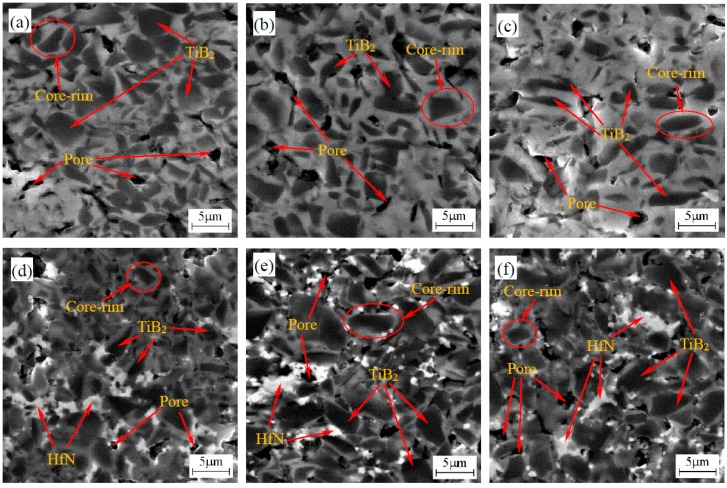
SEM-BSE photographs of the polished surfaces of TiB_2_-HfB_2_ and TiB_2_-HfN ceramic tool materials: (**a**) TiB_2_-10 wt % HfB_2_; (**b**) TiB_2_-20 wt % HfB_2_; (**c**) TiB_2_-30 wt % HfB_2_; (**d**) TiB_2_-10 wt % HfN; (**e**) TiB_2_-20 wt % HfN; (**f**) TiB_2_-30 wt % HfN.

**Figure 3:**
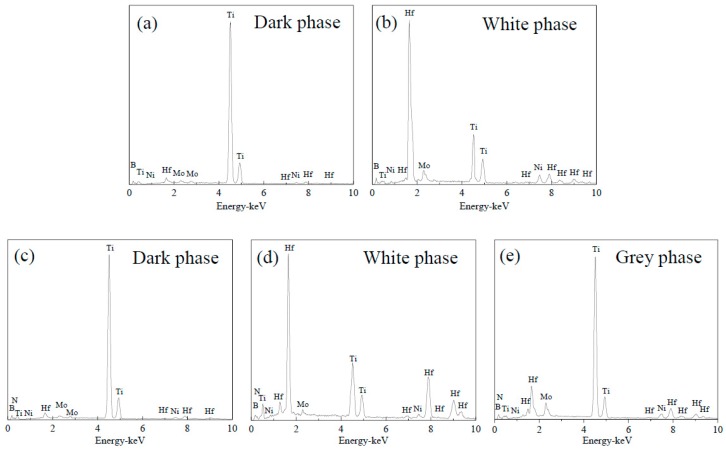
EDS of the phases in the TiB_2_-HfB_2_ ceramic tool materials: (**a**) EDS of the dark phase; (**b**) EDS of the white phase, and EDS of the phases in the TiB_2_-HfN ceramic tool materials; (**c**) EDS of the dark phase; (**d**) EDS of the white phase; (**e**) EDS of the grey phase.

SEM-BSE photographs of the polished surfaces of the TiB_2_-HfB_2_ and TiB_2_-HfN ceramic tool materials are presented in Figure 2. An obvious difference between the TiB_2_-HfB_2_ and TiB_2_-HfN ceramics is the presence of two phases (dark phase and white phase) in Figure 2a–c, while there are three phases (dark phase, white phase, and grey phase) in Figure 2d–f. The dark phase in Figure 2a–f is TiB_2_ based on the XRD and EDS results in Figure 3a and Figure 3c. The white phase in Figure 2a–c is mainly HfB_2_ according to the XRD and EDS results in Figure 3b, while the white phase in Figure 2d–f is mainly HfN according to the XRD and EDS results in Figure 3d. The grey phase in Figure 2d–f consists of TiB_2_ and HfN based on the XRD and EDS results in Figure 3e. Ni and Mo were also discovered in the EDS results in Figure 3. It is notable that the typical core-rim structures and pores exist in these ceramics as shown in Figure 2. The cores—the TiB_2_ grains—are wrapped by the rims. The rim phase in Figure 2a–c is composed of the Ni_3_Mo intermetallic compound and the complex solid solution of HfB_2_ and TiB_2_. However, the rim phase in Figure 2d–f is composed of the Ni_3_Mo intermetallic compound and the potential complex solid solution of HfN and TiB_2_. Moreover, in the TiB_2_-HfB_2_ ceramics, the rim phase (the complex solid solution of HfB_2_ and TiB_2_) gradually occupies a leading position as the HfB_2_ content increases. The number of pores decrease slightly in Figure 2a–c, but increase gradually in Figure 2d–f. In terms of size and shape, the pores in Figure 2a–c are bigger than that in Figure 2d–f; the regular pore shape in Figure 2a–c looks like the TiB_2_ grain shape and the irregular pore shape in Figure 2d–f looks like the shape of agglomerated HfN grains possibly pulled out in the grinding and polishing process, which indicates that a weaker grain boundary strength formed in these ceramics in the sintering processing. Moreover, in Figure 2a–c the morphology of some TiB_2_ grains changes from bigger polygons to smaller polygons or longer ovals which is advantageous for the formation of a relatively fine microstructure, and in Figure 2d–f the HfN grain agglomeration becomes more serious leading to the formation of more TiB_2_ coarse grains and pores. This indicates that the HfB_2_ additive not only can inhibit the growth of TiB_2_ grains but can also change the morphology of some TiB_2_ grains, and that the HfN additive exhibits a tendency toward agglomeration.

**Figure 4:**
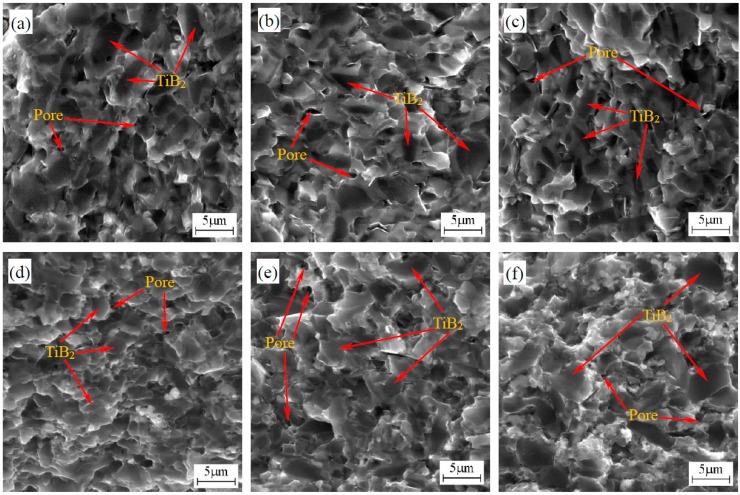
Fracture morphology of TiB_2_-HfB_2_ and TiB_2_-HfN ceramic tool materials: (**a**) TiB_2_-10 wt % HfB_2_; (**b**) TiB_2_-20 wt % HfB_2_; (**c**) TiB_2_-30 wt % HfB_2_; (**d**) TiB_2_-10 wt % HfN; (**e**) TiB_2_-20 wt % HfN; (**f**) TiB_2_-30 wt % HfN.

Figure 4 shows the fracture morphology of the TiB_2_-HfB_2_ and TiB_2_-HfN ceramic tool materials. As can be seen in Figure 4a–c, with increasing HfB_2_ content from 10 wt % to 30 wt %, the TiB_2_ grains become smaller; meanwhile, the TiB_2_ grain shapes exhibit the same variation trend as presented in Figure 3a–c; moreover, the pore number decreases progressively. However, in Figure 4d–f with increasing HfN content from 10 wt % to 30 wt %, the TiB_2_ grains become larger leading to the formation of coarse TiB_2_ grains; and the pore number decreases progressively. The results indicate that the HfB_2_ additive can not only inhibit the growth of the TiB_2_ grains, but can also change the microstructure of TiB_2_-based ceramic, and that the HfN additive cannot inhibit the TiB_2_ grain growth.

**Figure 5:**
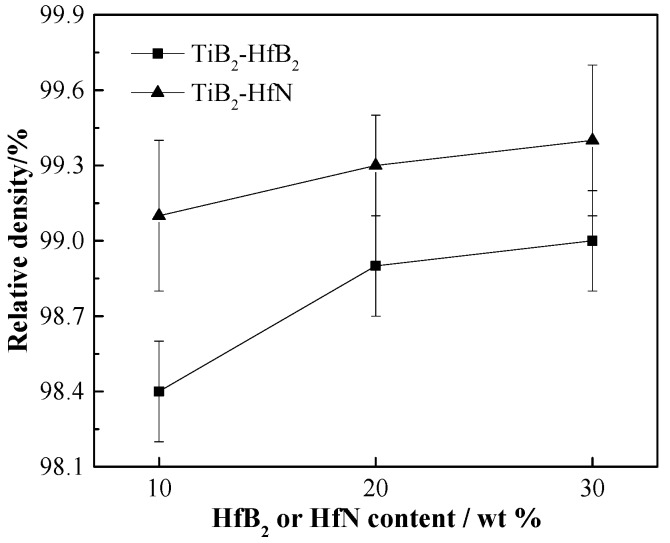
Relative densities of TiB_2_-HfB_2_ and TiB_2_-HfN ceramic tool materials.

Figure 5 presents the relative densities of the TiB_2_-HfB_2_ and TiB_2_-HfN ceramic tool materials. As can be seen, their relative densities increase with increasing HfB_2_ and HfN contents from 10 wt % to 30 wt %, respectively. The relative density increments of the TiB_2_-HfN ceramics are smaller, and the relative density variation curve is relatively flat, while the relative density variation curve of TiB_2_-HfB_2_ shows a bigger increment in relative density at first, and then finally shows a smaller increment. These results are ascribed to the pore number reduction with increasing the additive content, to some extent, and is derived from the higher sintering pressure (30 MPa) and the metal phases (Ni and Mo) that can efficiently reduce the sintering temperature and can accelerate the densification of these ceramics. As a consequence, their relative densities with the addition of HfB_2_ or HfN can be improved, and when the HfB_2_ and HfN contents are 30 wt %, the optimal relative densities of the TiB_2_-HfB_2_ and TiB_2_-HfN ceramics are 99.0% ± 0.2% and 99.4% ± 0.3%, respectively.

### 3.2. Mechanical Properties

**Figure 6:**
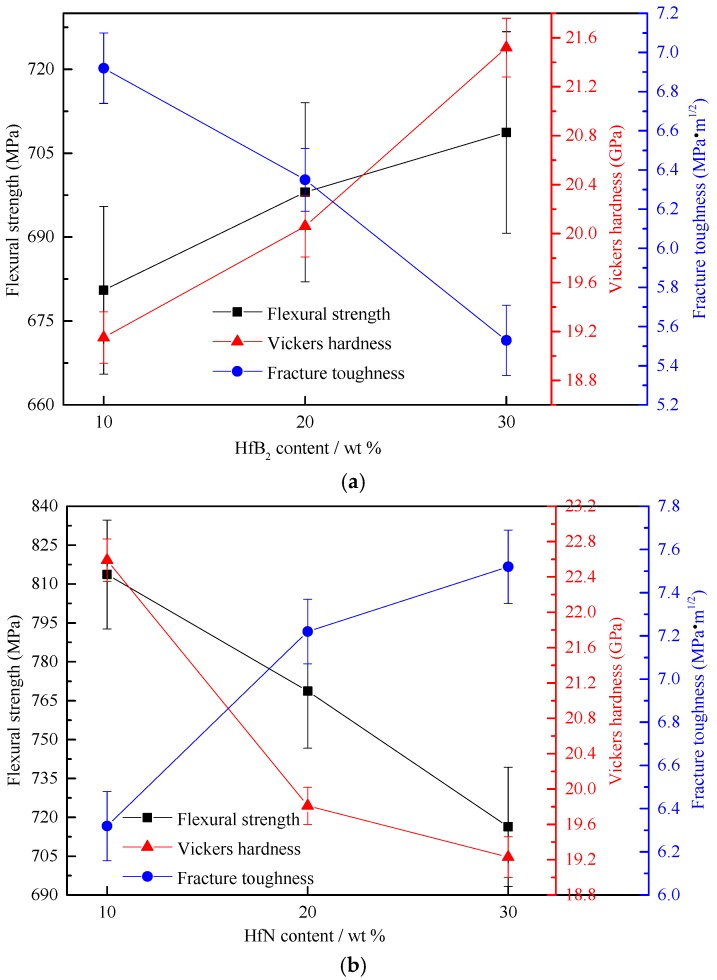
Variation of the mechanical properties of TiB_2_-HfB_2_ ceramics with a change of the HfB_2_ content and variation of the mechanical properties of TiB_2_-HfN ceramics with a change of the HfN content.

Figure 6 exhibits the variation of the mechanical properties of the TiB_2_-HfB_2_ ceramics with changes of the HfB_2_ content and variation of the mechanical properties of the TiB_2_-HfN ceramics with changes of the HfN content. In Figure 6a, with the increase of the HfB_2_ content from 10 wt % to 30 wt %, the flexural strength increases from 680.49 ± 15 MPa to 708.71 ± 18 MPa; Vickers hardness increases from 19.15 ± 0.21 GPa to 21.52 ± 0.24 GPa; however, the fracture toughness decreases from 6.92 ± 0.18 MPa·m^1/2^ to 5.53 ± 0.18 MPa·m^1/2^. The TiB_2_-30 wt %HfB_2_ ceramic tool material exhibits better mechanical properties including flexural strength of 708.71 ± 18 MPa, which is higher than 533 MPa (the flexural strength of TiB_2_-TaC ceramics [9]), Vickers hardness of 21.52 ± 0.24 GPa that is higher than 19.8 ± 0.6 GPa (Vickers hardness of the TiB_2_-SiC-CNTs ceramics [8]), and fracture toughness of 5.53 ± 0.18 MPa·m^1/2^ that is higher than 5.2 MPa·m^1/2^ (fracture toughness of the TiB_2_-SiC ceramics [31]). The improvement of flexural strength and Vickers hardness is due to the relatively fine microstructure, which is in agreement with the result that the fine microstructure can improve the mechanical properties of ceramic composite materials [32]. As the HfB_2_ content increases, the fracture toughness decreases gradually, which can be ascribed to the increase of the brittle rim phase. The reason is that the rim phase is mainly the complex solid solution of TiB_2_ and HfB_2_, which may be a brittle phase; moreover, the wettability between HfB_2_ and Ni (the wettability angle: ~99°) is poor.

In Figure 6b, with the increase of the HfN content from 10 wt % to 30 wt %, the flexural strength decreases from 813.69 ± 21 MPa to 716.37±23 MPa; the Vickers hardness decreases from 22.59 ± 0.24 GPa to 19.23 ± 0.23 GPa; however, the fracture toughness increases from 6.32 ± 0.16 MPa·m^1/2^ to 7.52 ± 0.17 MPa·m^1/2^. The TiB_2_-10 wt % HfN ceramic tool material shows better mechanical properties, including flexural strength of 813.69 ± 21 MPa that is higher than 705 MPa (flexural strength of the TiB_2_-10 wt % SiC ceramics [6]), Vickers hardness of 22.59 ± 0.24 GPa which is higher than 21.85 GPa (Vickers hardness of the TiB_2_-TiC-10 wt % Ni ceramics [33]), and fracture toughness of 6.32 ± 0.16 MPa·m^1/2^ that is higher than 6 MPa·m^1/2^ (fracture toughness of the TiB_2_-2.5 wt % MoSi ceramics [15]). The decrease of the flexural strength and Vickers hardness is due to the increase of the defects such as the TiB_2_ coarse grain and HfN agglomeration; this indicates that the defects have more negative effects on the flexural strength and Vickers hardness than the core-rim structure, although the core-rim structure is advantageous for improving the mechanical properties. The enhancement of fracture toughness is mainly attributed to the decrease of the pore number and the increase of the rim phase and TiB_2_ coarse grain; decreasing the pore formation can keep the cracks from growing, which will improve fracture toughness; the rim phase of TiB_2_-HfN ceramics exhibits a higher grain boundary strength than the rim phase of TiB_2_-HfB_2_ ceramics, which will provide a larger grain growth resistance for enhancing fracture toughness; in addition, TiB_2_ coarse grains can consume more fracture energy in the fracturing process even though the TiB_2_ is a brittle phase, which leads to the improvement of the fracture toughness.

**Figure 7:**
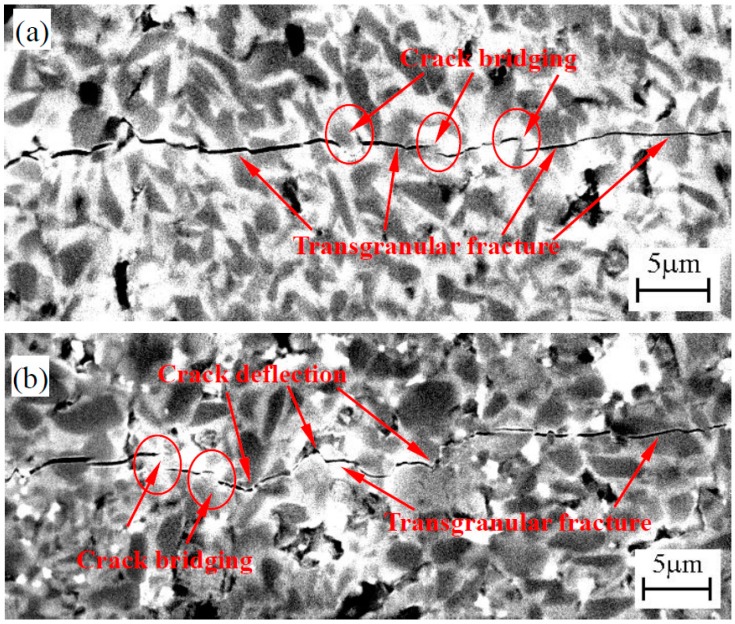
Crack propagation path of the TiB_2_-HfB_2_ (**a**) and TiB_2_-HfN (**b**) ceramic tool materials.

In order to further analyze the toughening mechanisms of TiB_2_-HfB_2_ and TiB_2_-HfN ceramics, the crack propagation paths are shown in Figure 7. As can be seen, the crack propagation path in Figure 7a is straighter than that in Figure 7b; the crack deflection in Figure 7b is more obvious than that in Figure 7a; crack bridging and transgranular fracture play an important role in Figure 7a, while crack deflection, crack bridging, and transgranular fracture occupy important positions in Figure 7b, which are advantageous for enhancing fracture toughness and are the main toughening mechanisms of these ceramics. Much fracture energy will be consumed by crack bridging because crack bridging as well as crack deflection can change the direction of crack propagation (see the red circles in Figure 7), which is advantageous for improving fracture toughness. Usually the formation of the rim phase is propitious to the enhancement of fracture toughness, but in Figure 7a the rim phase shows a brittle characteristic leading to lower fracture toughness with increasing HfB_2_ content as mentioned above; moreover, the relatively straight crack crossing the rim phase and TiB_2_ grain will consume less fracture energy, which is harmful to the improvement of fracture toughness. However, intergranular fracture and transgranular fracture coexisted in Figure 7b, where the crack path is full of twists and turns which is advantageous to enhancing fracture toughness.

## 4. Conclusions

TiB_2_-based ceramic tool materials reinforced by HfB_2_ and HfN additives have been fabricated by hot pressed sintering. The effects of HfB_2_ and HfN additions on their microstructures and mechanical properties were investigated. The results showed that the HfB_2_ additive can inhibit the TiB_2_ grain growth and can change the morphology of some of the TiB_2_ grains from bigger polygons to smaller polygons or longer ovals, which is favorable for the formation of a relatively fine microstructure, while the HfN additive tends to agglomerate. With increasing HfB_2_ and HfN contents from 10 wt % to 30 wt %, the relative densities of these ceramics increased gradually. The relatively fine microstructure improved the flexural strength and Vickers hardness of the TiB_2_-HfB_2_ ceramics. The poor wettability between HfB_2_ and Ni resulted in the formation of weak grain boundary strength and the complex solid solution of TiB_2_-HfB_2_ is a brittle phase, which led to the decrease of fracture toughness of the TiB_2_-HfB_2_ ceramics. The increase of the defects such as the TiB_2_ coarse grain and HfN agglomeration resulted in the decrease of the flexural strength and Vickers hardness of the TiB_2_-HfN ceramics; the decrease of the pore number and the increase of the rim phase and TiB_2_ coarse grain are advantageous for the enhancement of fracture toughness. The toughening mechanisms of the TiB_2_-HfB_2_ ceramics mainly included crack bridging and transgranular fracture, while the toughening mechanisms of the TiB_2_-HfN ceramics mainly included crack deflection, crack bridging, transgranular fracture, and the core-rim structure. The TiB_2_-30 wt % HfB_2_ ceramic tool material exhibited better mechanical properties including a flexural strength of 708.71 ± 18 MPa, Vickers hardness of 21.52 ± 0.24 GPa, and fracture toughness of 5.53 ± 0.18 MPa·m^1/2^. The TiB_2_-10 wt % HfN ceramic tool material showed better mechanical properties including a flexural strength of 813.69 ± 21 MPa, Vickers hardness of 22.59 ± 0.24 GPa, and fracture toughness of 6.32 ± 0.16 MPa·m^1/2^.

## Figures and Tables

**Table 1:** Compositions of TiB_2_-HfB_2_ and TiB_2_-HfN ceramic tool materials.

Sample	TiB_2_/wt %	HfB_2_/wt %	HfN/wt %	Ni/wt %	Mo/wt %
S1	82	10	-	4	4
S2	72	20	-	4	4
S3	62	30	-	4	4
S4	82	-	10	4	4
S5	72	-	20	4	4
S6	62	-	30	4	4

## References

[1] Wang M., Zhao J., Wang L.L. (2015). Wear behaviour of Al_2_O_3_/Ti(C,N) ceramic tool during turning process of martensitic stainless steel. Mater. Res. Innov..

[2] Zheng G.M., Zhao J., Zhou Y.H., Li A.H., Cui X.B., Tian X.H. (2013). Performance of graded nano-composite ceramic tools in ultra-high-speed milling of Inconel 718. Int. J. Adv. Manuf. Technol..

[3] Wang D., Zhao J., Cao Y., Xue C., Bai Y. (2016). Wear behavior of an Al_2_O_3_/TiC/TiN micro-nano-composite ceramic cutting tool in high-speed turning of ultra-high-strength steel 300 M. Int. J. Adv. Manuf. Technol..

[4] Kwon W.T., Kim Y.W. (2004). Cutting performance of Si_3_N_4_ based SiC ceramic cutting tools. J. Mech. Sci. Technol..

[5] Song J.P., Huang C.Z., Lv M., Zou B., Liu H.L., Wang J. (2014). Cutting performance and failure mechanisms of TiB_2_-based ceramic cutting tools in machining hardened Cr_12_MoV mold steel. Int. J. Adv. Manuf. Technol..

[6] Zhao G.L., Huang C.Z., He N., Liu H.L., Zou B. (2016). Preparation and cutting performance of reactively hot pressed TiB_2_-SiC ceramic tool when machining Invar36 alloy. Int. J. Adv. Manuf. Technol..

[7] Vlasova1 M., Bykov A., Kakazey M., Aguilar P.A.M., Melnikov I., Rosales I., Tapia R.G. (2016). Formation and Properties of TiB_2_-Ni Composite Ceramics. Sci. Sinter..

[8] Lin J., Yang Y.H., Zhang H.A., Chen W.F., Huang Y. (2016). Microstructure and mechanical properties of TiB_2_ ceramics enhanced by SiC particles and carbon nanotubes. Ceram. Int..

[9] Demirskyi D., Nishimura T., Sakka Y., Vasylkiv O. (2016). High-strength TiB_2_-TaC ceramic composites prepared using reactive spark plasma consolidation. Ceram. Int..

[10] Raju G.B., Basu B., Tak N.H., Cho S.J. (2009). Temperature dependent hardness and strength properties of TiB_2_ with TiSi_2_ sinter-aid. J. Eur. Ceram. Soc..

[11] Popov A.Y., Sivak A.A., Borodianska H.Y., Shabalin I.L. (2015). High toughness TiB_2_-Al_2_O_3_ composite ceramics produced by reactive hot pressing with fusible components. Adv. Appl. Ceram..

[12] Song J.P., Huang C.Z., Lv M., Zou B., Wang S.Y., Wang J., An J. (2014). Effects of TiC content and melt phase on microstructure and mechanical properties of ternary TiB_2_-based ceramic cutting tool materials. Mater. Sci. Eng. A.

[13] Gao Y.B., Tang T.G., Yi C.H., Zhang W., Li D.C., Xie W.B., Huang W., Ye N. (2016). Study of static and dynamic behavior of TiB_2_-B_4_C composite. Mater. Des..

[14] Demirskyi D., Sakka Y., Vasylkiv O. (2015). High-temperature reactive spark plasma consolidation of TiB_2_-NbC ceramic composites. Ceram. Int..

[15] Mukhopadhyay A., Raju G.B., Basu B., Suri A.K. (2009). Correlation between phase evolution, mechanical properties and instrumented indentation response of TiB_2_-based ceramics. J. Eur. Ceram. Soc..

[16] Li B. (2014). Effect of ZrB_2_ and SiC addition on TiB_2_-based ceramic composites prepared by spark plasma sintering. Int. J. Refract. Met. Hard Mater..

[17] Sonber J.K., Murthy T.S.R.C., Subramanian C., Krishnamurthy N., Hubli R.C., Suri A.K. (2012). Effect of CrSi_2_ and HfB_2_ addition on densification and properties of ZrB_2_. Int. J. Refract. Met. Hard Mater..

[18] Balak Z., Zakeri M. (2016). Effect of HfB_2_ on microstructure and mechanical properties of ZrB_2_-SiC-based composites. Int. J. Refract. Met. Hard Mater..

[19] Tu R., Li N., Li Q.Z., Zhang S., Zhang L.M., Goto T. (2016). Effect of microstructure on mechanical, electrical and thermal properties of B_4_C-HfB_2_ composites prepared by arc melting. J. Eur. Ceram. Soc..

[20] Wang S.H., Zhang Y.C., Sun Y., Xu Y., Yang M. (2016). Synthesis and characteristic of SiBCN/HfN ceramics with high temperature oxidation resistance. J. Alloys Compd..

[21] Li B.H., Liu Y., Cao H., He L., Li J. (2009). Rapid synthesis of TiB_2_/Fe composite in situ by spark plasma sintering. J. Mater. Sci..

[22] Sáez A., Arenas F., Vidal E. (2003). Microstructure development of WCoB-TiC based hard materials. Int. J. Refract. Met. Hard Mater..

[23] Zhang G., Yang J.H. (2013). In situ synthesis aluminum borate whiskers reinforced TiB_2_ matrix composites for application in aluminum reduction cells. JOM.

[24] Farhadi K., Namini A.S., Asl M.S., Mohammadzadeh A., Kakroudi M.G. (2016). Characterization of hot pressed SiC whisker reinforced TiB_2_ based composites. Int. J. Refract. Met. Hard Mater..

[25] Cui H.Z., Zhang Y.F., Zhang G.S., Liu W., Song X.J., Wei N. (2016). Pore and microstructure change induced by SiC whiskers and particles in porous TiB_2_-TiC-Ti_3_SiC_2_ composites. Ceram. Int..

[26] China State Bureau of Technological Supervision (2006). Chinese National Standards—Fine Ceramics (Advanced Ceramics, Advanced Technical Ceramics)—Test Method for Flexural Strength of Monolithic Ceramics at Room Temperature.

[27] Zhao G.L., Huang C.Z., He N., Liu H.L., Zou B. (2016). Microstructure and mechanical properties at room and elevated temperatures of reactively hot pressed TiB_2_-TiC-SiC composite ceramic tool materials. Ceram. Int..

[28] China State Bureau of Technological Supervision (2009). Chinese National Standards—Fine Ceramics (Advanced Ceramics, Advanced Technical Ceramics)—Test Method for Flexural Strength of Monolithic Ceramics at Room Temperature.

[29] Khalfallah I., Aning A. Bulk Processing and Mechanical Properties of Ni_3_Mo. Proceedings of the 143rd TMS Annual Meeting & Exhibition.

[30] Qi L., Jin Y.C., Zhao Y.H., Yang X.M., Zhao H., Han P. (2015). The structural, elastic, electronic properties and Debye temperature of Ni_3_Mo under pressure from first-principles. J. Alloys Compd..

[31] Chen H.B., Wang Z., Wu Z.J. (2014). Investigation and characterization of densification, processing and mechanical properties of TiB_2_-SiC ceramics. Mater. Des..

[32] Anggraini L., Isonishi K., Ameyama K. (2016). Toughening and strengthening of ceramics composite through microstructural refinement. Am. Inst. Phys..

[33] Fu Z.Z., Koc R. (2016). Sintering and mechanical properties of TiB_2_-TiC-Ni using submicron borides and carbides. Mater. Sci. Eng. A.

